# Effects of therapeutic hypercapnia on the expression and function of γδT cells in transplanted lungs in rats

**DOI:** 10.1002/iid3.1220

**Published:** 2024-03-20

**Authors:** Qing Xie, Jia Lu, XiaoGuang Cui

**Affiliations:** ^1^ Department of Anesthesia, First Affiliated Hospital Zhejiang University School of Medicine Hangzhou China; ^2^ Department of Anesthesia, Huashan Hospital, Shanghai Medical College Fudan University Shanghai China; ^3^ Department of Anesthesia First Affiliated Hospital of Hainan Medical College Haikou Hainan China

**Keywords:** hypercapnia, IL‐17, ischemia‐reperfusion injury, lung transplantation, γδT cells

## Abstract

**Objective:**

To investigate the effect of therapeutic hypercapnia on the expression and function of gamma delta T (γδ T) cells during ischemia‐reperfusion injury (IRI) after lung transplantation.

**Methods:**

We randomly divided male Wistar rats into three groups (*n* = 6 in each group), the control group (group N), the IRI group (group I), and the therapeutic hypercapnia group (group H). We then assessed pulmonary edema, neutrophil infiltration, wet‐to‐dry (W/D) weight ratio, and microscopic histopathology and separately measured the levels of γδT cell surface antigen (TCR) and Interleukin‐17 (IL‐17) using flow cytometry and enzyme‐linked immunosorbent assays (ELISAs).

**Results:**

The infiltration of neutrophils and the expression of TCR and IL‐17 were significantly increased in the I group compared to the control, and the biopsy edema in group I was more severe. Arterial partial pressure of oxygen (PaO2) was decreased after reperfusion in group I compared with the control group. W/D weight ratio, neutrophil infiltration, and the expression of TCR and IL‐17 decreased drastically in the H group compared to the I group.

**Conclusion:**

Our findings suggest that γδ T lymphocytes were directly involved in lung injury. In addition, therapeutic hypercapnia effectively reduced the expression of γδ T cells and IL‐17, and this has the potential to become a treatment strategy for IRI and an intervention to improve lung function.

## Graphical abstract

We hypothesized that hypercapnia could reduce γδ T cell expression, and we explored how hypercapnia regulates γδ T cell expression to attenuate ischemia‐reperfusion injury in lung transplantation.

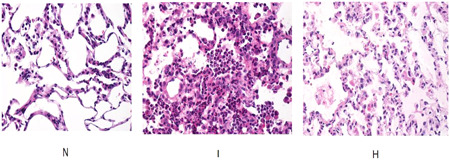

## 1 INTRODUCTION

Lung transplantation has become an effective treatment for end‐stage lung diseases and has been in clinical use since 1983.^1^ Currently, the mortality caused by lung ischemia‐reperfusion injury (IRI) post‐surgery is estimated to be 16%–25% and has attracted attention globally.^2^, ^3^ Thus, studying the mechanism of lung IRI and identifying effective strategies to reduce mortality by protecting lung function has gained much significance.

In recent years, researchers have focused on the role of T lymphocytes, which have shown remarkable effects during lung IRI.^4^, ^5^ Gamma delta T (γδT) cells, which account for less than 5% of the total number of T lymphocytes, have gradually become a new popular topic.^6^, ^7^ Relatively little is known about the expression and function of γδT cells in transplanted lungs in rats. It has been found that the multiplication of γδT cells seriously affects the homeostasis of polymorphonuclear leukocytes (PMNs), at least in part, resulting in simultaneous neutrophil infiltration and increased Interleukin‐17 (IL‐17) secretion.^8^ Studies have indicated that γδT cells are also involved in renal IRI.^9^ In cerebral IRI, γδT cells and secreted IL‐17 were shown to be involved in IRI.^10^, ^11^ In our earlier study, we found that IL‐17A, produced primarily by γδT cells, played a pathogenic role in myocardial IRI by inducing cardiomyocyte apoptosis and neutrophil infiltration.^8^ However, whether γδT cells play a major role in lung IRI after transplantation is still unknown.

Many studies have shown that therapeutic hypercapnia has protective effects on lung IRI,^12^, ^13^ but its specific mechanism of action is still not fully understood. Current studies have demonstrated that therapeutic hypercapnia can alleviate lung IRI by regulating the expression of T lymphocytes.^14^ Previously, we have described that γδT cells participate in various IRIs, but whether hypercapnia has an effect on γδT cells is still unclear. Therefore, we hypothesized that hypercapnia could reduce γδ T cell expression, and we explored how hypercapnia regulates γδ T cell expression to attenuate IRI in lung transplantation.

## 2 MATERIALS AND METHODS

### 2.1 Animals

We obtained 18 8 to 10‐week‐old male Wistar rats (250–300 g) from Charles River Laboratories bred in the Harbin Medical University animal facilities. All rats had permissive access to food and water under controlled conditions 12/12 h light/dark with humidity of 50 ± 5%, 22 ± 4°C).

### 2.2 Antibodies and drugs

We used pancreatic enzymes and collagenase to digest lung tissue. Red Blood Cell Lysis Buffer, obtained from Becton, Dickinson and Company (B&D) Systems, was used to lyse red blood cells in the filtrate of digested lung slices. We obtained PerCP‐Cyanine 5.5(PerCP/Cy5.5) antirat CD45, phycoerythrin (PE) mouse antirat γδT cell receptor, and PE mouse antirat IgG receptor from B&D Systems to assess γδT cells. Rat IL‐17 enzyme‐linked immunosorbent assay (ELISA) kits were bought from BEINGLAY Biotech Co., Ltd., Wu Han, and were used to detect the expression of IL‐17.

### 2.3 Experimental design

We randomly divided male Wistar rats into three groups, (*n* = 6 in each group) the control group N (open thoracic surgery only for 1 h and euthanized after 24 h), the IRI group I (1 h ischemia and 24 h reperfusion including a mechanical ventilation gas mixture of 50% oxygen [O_2_] and 50% nitrogen [N_2_] for the first hour), and the therapeutic hypercapnia group H (1 h ischemia and 24 h reperfusion including mechanical ventilation of a CO_2_ and O_2_ mixture to reach a partial pressure of arterial carbon dioxide [PaCO2] of 80–100 mmHg for the first hour). Due to the collection of different kinds of specimens (the process of obtaining bronchoalveolar lavage fluid [BALF] can affect the alveolar structure and the measurement of γδT cells and neutrophils in alveoli), the experiments were repeated twice.

### 2.4 Lung transplantation model

The establishment of donor lung model: Establishment of the orthotopic lung transplantation rat model was done as per Mizuta's method.^15^ In general, the donor rat was anesthetized using intraperitoneal injection with 30 mg/kg of pentobarbital hydrochloride, and 300 units of heparin were injected into the rat tail vein for systemic heparinization. Then tracheostomy was done for volume‐controlled mechanical ventilation.

About 5 min after heparinization, the chest and abdomen were disinfected with iodophor, and the rat's fur was cut in an inverted “T” shape below the xiphoid. A median sternotomy was performed, and the lung lobe was carefully protected from accidental injury during the thoracotomy process. We used two forceps to clamp the edges of the sternum to open the chest cavity. While carefully clamping the heart with forceps in the left hand, a small incision was made in the left and right auricular appendages and the right ventricle with the scissors in the right hand.

Air was expelled from the lavage device containing 20 mL low potassium dextran (LPD) lavage solution at 4°C, and then the 14 gauge (14G) deep vein indwelling needle was inserted as the tube head of the lavage device through the right ventricle to the pulmonary trunk. The indwelling needle was fixed with arterial clamps. Under pressure of 20 cm water column (cmH_2_O), the chest cavity was flushed with normal saline at 4°C to maintain a low‐temperature environment while irrigating. Lavage was done until both lungs were white. The trachea was ligated when inflating air into the lungs, and the trachea was cut off above the ligation. The esophagus was separated, and the lungs and heart as a whole were removed. Then the whole lungs were placed under the light microscope, and the cuff cannula was inserted into the donor pulmonary artery, pulmonary vein, and trachea.

During the procedure, the left lung was flushed with 4°C normal saline to prevent water loss and drying under the microscope, to save time, and minimize damage to the donor lung induced by the procedure and the like. The trachea was then clamped with arterial clamping when the left lung was semi‐inflated and stored at 4°C with wet yarn.

Establishment of the receptor orthotopic lung transplantation model: The recipient rat was anesthetized and intubated with a 14G tracheal tube while the cuff was placed in the left lung. The rat's tail artery was punctured, and a catheter was inserted. A monitor was connected for blood pressure detection and arterial blood gas analysis. The recipient rat was placed lying on its right side, and the skin of the left chest was prepared and disinfected. After suction, a ventilator was connected for mechanical ventilation, with a tidal volume of 8–10 mL/kg, frequency of 55–60 times/min, and inhaled oxygen concentration of 50%. The skin was disinfected with iodophor for preparation.

The chest was opened at the rib margin at the level where the cardiac pulsation was most visible (approximately the fifth intercostal space), and a pull hook was used to open the chest cavity and expose the surgical field. After the left lung was removed and fixed with a lung clip, the hilar was exposed under the microscope, and the left lung arteriovenous and left bronchi were isolated and clamped at the proximal end with an arterial clip. Then the left lung was carefully removed. The residual air and fluid in the donor's lung were sucked out and anastomosed with the corresponding structures of the recipient in the order of the artery, left main trachea, and vein.

After cuff anastomosis, a cotton ball was used to remove the exudate or effusion in the pleural cavity, and the vein left main bronchus, and artery were opened sequentially. If the lung was well‐inflated, the blood flow in the lung was smooth and the lung tissue quickly turned pink, and there was no blood leakage or exudation at the three anastomotic locations, it indicated that the transplantation was successful. The left lung was placed back into the chest cavity, and 2 cmH_2_O of positive end‐expiratory pressure (PEEP) was continuously administered to prevent lung collapse and atelectasis. The chest wall was sutured, and arterial blood gas analysis was done at the specified time point.

The rat was awakened and extubated after about 1 h; it was euthanized 24 h later and the experimental specimens were obtained. The samples include tissues (the lung was divided into three segments: the upper segment was used for dry–wet ratio measurement, the middle segment was used for hematoxylin‐eosin [HE] staining and neutrophil counting, and the lower segment was used for cell collection and T cell receptor [TCR] measurement of γδT cells), lavage solution, and blood samples which were used for detecting IL‐17.^16^

### 2.5 Blood samples for blood gas analysis

Arterial blood samples were collected before the procedure and at 15, 30, and 45 min during left lung reperfusion, and we performed blood gas analysis, such as pH, PaO2, and PaCO2 analyses, to evaluate and control the rats' conditions during the experiment.

### 2.6 Bronchoalveolar lavage (BAL) and serum

The left lower lobe bronchus of the control group, IRI group, and TH group was cannulated via an endotracheal tube (ETT) tube and lavaged with 9 mL of saline (0.9%), and then, 5–7 mL of BALF was collected. About 8–9 mL of blood was collected from the femoral artery. The BALF and blood samples were centrifuged at 3000 rotation per minute (rpm) at 4°C for 15 min, and the supernatant was divided into 1.5 mL aliquots and stored at −80°C for subsequent assays.

### 2.7 Wet‐to‐dry (W/D) weight ratio

The remainder of the left upper lobe lung was utilized for determining the lung W/D weight ratio after sequential weighs demonstrated maximal dehydration in a drying oven.

### 2.8 Histologic examination

The middle sections of the left lungs of the rats were obtained from the three groups, submerged in 10% formalin in neutral buffered solution for immediate fixation, and later embedded in paraffin. The lung tissues were stained with hematoxylin and eosin (H&E), dehydrated, and slipped in a cover. Morphologic examination was performed using OLYMPUS light microscopy (manufactured in Japan) and documented using photographs.^16^

### 2.9 Measurement of the inflammatory cytokine IL‐17

The BALF and serum from recipients were centrifuged at 3000 rpm at 4°C for 15 min. IL‐17 was measured using an ELISA, following the procedure described by the manufacturer.^17^

### 2.10 Identification and enumeration of γδT cells

(1) The lower segment of the recipient's left lung was taken out and placed in a flat‐bottomed glassware. The glassware was placed on ice, and the lung tissue was cut into pieces with small scissors. The appropriate amount of phosphate‐buffered saline (PBS) liquid was added to soak the lung tissue until the lung tissue could be fully suspended in the liquid. A sucker was used to transfer it into a 10 mL centrifuge tube.
(2) The liquid was centrifuged at a speed of 1500 rpm for 15 min, and the supernatant was discarded.
(3) Six milliliters of trypsin and collagenase I that was prepared in a sterile environment half an hour earlier were added into the precipitate and mixed well in a diagonal glass culture vessel. The mixture was shaken horizontally for 1 h in a water bath shaker at 37°C, with 200 revolutions per minute, and the mixture was thoroughly pipetted and mixed well every 30 min.
(4) After 1 h, the digested lung tissue was filtered with a 300‐mesh filter into a 10 mL centrifuge tube. The tube was centrifugated at 1500 rpm for 15 min after balancing. The supernatant was discarded.
(5) Two milliliters of red blood cell lysate was added to the substrate and placed in the dark for 10 min. Then it was centrifuged at 2500 rpm for 10 min. The above process was repeated twice with PBS solution to obtain lung tissue cells.
(6) The lung tissue cells were resuspended in 1 mL of PBS solution and mixed well. One hundred microliters of liquid was extracted for the flow cytometry and divided into four groups. Five microliters of different antibodies were added into each group: Group A was the blank group, only PerCP/Cy5.5 antirat CD45 antibody (B&D, the same below) was added into group B, PE Mouse antirat IgG antibody (B&D) and CD45 antibody were added into group C, PE Mouse antirat γδT cell antibody (B&D) and CD45 antibody were added into group D. These four tubes were incubated in the dark at 4°C for 15‐30 min. The solution was diluted to 500 uL with PBS and detected and analyzed using a BD Aria flow cytometer (B&D). The specific settings were as follows: light source: PerCP/Cy5.5, excitation color was red, excitation wavelength was 488 nm, fluorescence channel was 690/50, PE excitation color was yellow, excitation wavelength was 488 nm, fluorescence channel was 585/42; gating strategy: the first step was to remove the cell debris, adherent bodies, and nonliving cells from the lung tissue cell suspensions, and the second step was to select the CD45+ and γδ TCR cells double gated, IgG TCR was done as isotype control.^16^

### 2.11 Neutrophil counting

The number of neutrophils was quantified using light microscopy. For each animal, the total tissue area was stained using the H&E method. We randomly selected five fields of a section from each rat to count neutrophils and calculated the mean number in one field to assess neutrophil infiltration.^16^

### 2.12 Statistical analysis

Data were expressed as mean ± standard deviation (SD). Statistical differences between group means were determined with one‐way or two‐way repeated‐measures analysis of variance followed by a post hoc comparison using the Newman–Keuls test. Comparisons within each group for a given parameter were performed using paired Student's *t* tests. Statistical significance was determined at *p* < .05 level.

## 3 RESULTS

### 3.1 Arterial blood gases and acid base data

The levels of PaO2, PaCO2, and pH were comparable in the three groups. Before reperfusion, there was no significant difference among these groups, and the basic levels were: PaO2 (150–180), PaCO2 (35–45), and pH (7.35–7.45) with mechanical ventilation of a mixture gas of approximately 50% O_2_ and 50% N_2_. Following reperfusion, PaCO2 (80–100) increased, pH (7.1–7.2) decreased progressively for the duration of the experiment in the H group, while PaO2 (180–200) increased significantly and was much higher than the baseline value. In the IRI group, PaCO2 increased and pH decreased, and PaO2 (90–110) reduced sharply during the procedure. The data showed that these indicators were stable in the control group (Table 1).

**Table 1:** Arterial blood gases and acid base data and Lung W/D weight ratio.

	N	I	H
PaO_2_ mmHg			
Baseline	174.2 ± 7.2	170.6 ± 18.2	163.9 ± 8.1
Final	163.6 ± 6.9	106.2 ± 5.4**	188.02 ± 25.4*
PaCO_2_ mmHg			
Baseline	39.2 ± 8.5	33.5 ± 3.2	35.9 ± 3.1
Final	35.5 ± 5.1	37.3 ± 2.3	86.95 ± 3.5*
PH			
Baseline	7.35 ± 0.02	7.38 ± 0.04	7.35 ± 0.02
Final	7.36 ± 0.04	7.25 ± 0.03*	7.12 ± 0.07**
W/D g	5.48 ± 0.14	6.45 ± 0.09**	5.71 ± 0.15*

*Note*: N: Control group, sham surgery only; I: Chemia reperfusion injury group; H: Hypercapnia group. Baseline: The data before operation. Final: The data at 30 min after reperfusion.

Abbreviations: PH, pondus hydrogenii; W/D, wet‐to‐dry.

* The mean difference is significant at the 0.05 level.

** The mean difference is significant at the 0.01 level.

### 3.2 Measurement of IL‐17 in BALF and serum

IL‐17 in the BALF and serum were elevated in the IRI group after 24 h. BALF levels of IL‐17 in rats with the transplanted lung were significantly higher than those in the control group (23.94 ± 1.76 vs. 19.89 ± 2.05; *p* < .05). Similarly, serum levels of IL‐17 in the IRI group were also significantly higher than those in the control group (25.56 ± 5.07 vs. 19.10 ± 1.86; *p* < .05).

**Figure 1:**
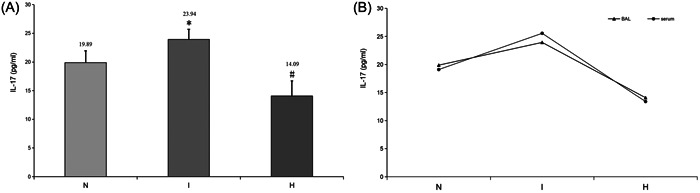
(A) BALF IL‐17 level as measured by ELISA. IL‐17 concentrations are elevated in the I group versus the control group and drop sharply in the H group. Data are expressed as the mean ± SD, bars represent SD, *p < .05, #*p* < .01, and six rats per group were examined. (B) There is a significant correlation between IL‐17 in BALF and serum. They have the same increasing trend in the three groups.

In addition, there was a notable correlation between serum and BALF concentrations in individual transplanted rats (*p* < .01). The IL‐17 concentrations in both BALF and serum showed the same increasing trend. Furthermore, BALF concentrations of IL‐17 in transplanted rats after CO_2_ treatment decreased rapidly and were much lower than those in the IRI group (14.09 ± 2.60 vs. 23.94 ± 1.76; *p* < .01) and even lower than those in the controls (*p* < .05). IL‐17 levels in the serum showed similar kinetics to those observed in BALF (13.35 ± 6.32 vs. 25.56 ± 5.07; *p* < .01) (Figure 1).

### 3.3 Accumulation of neutrophils in lung tissues

**Figure 2:**
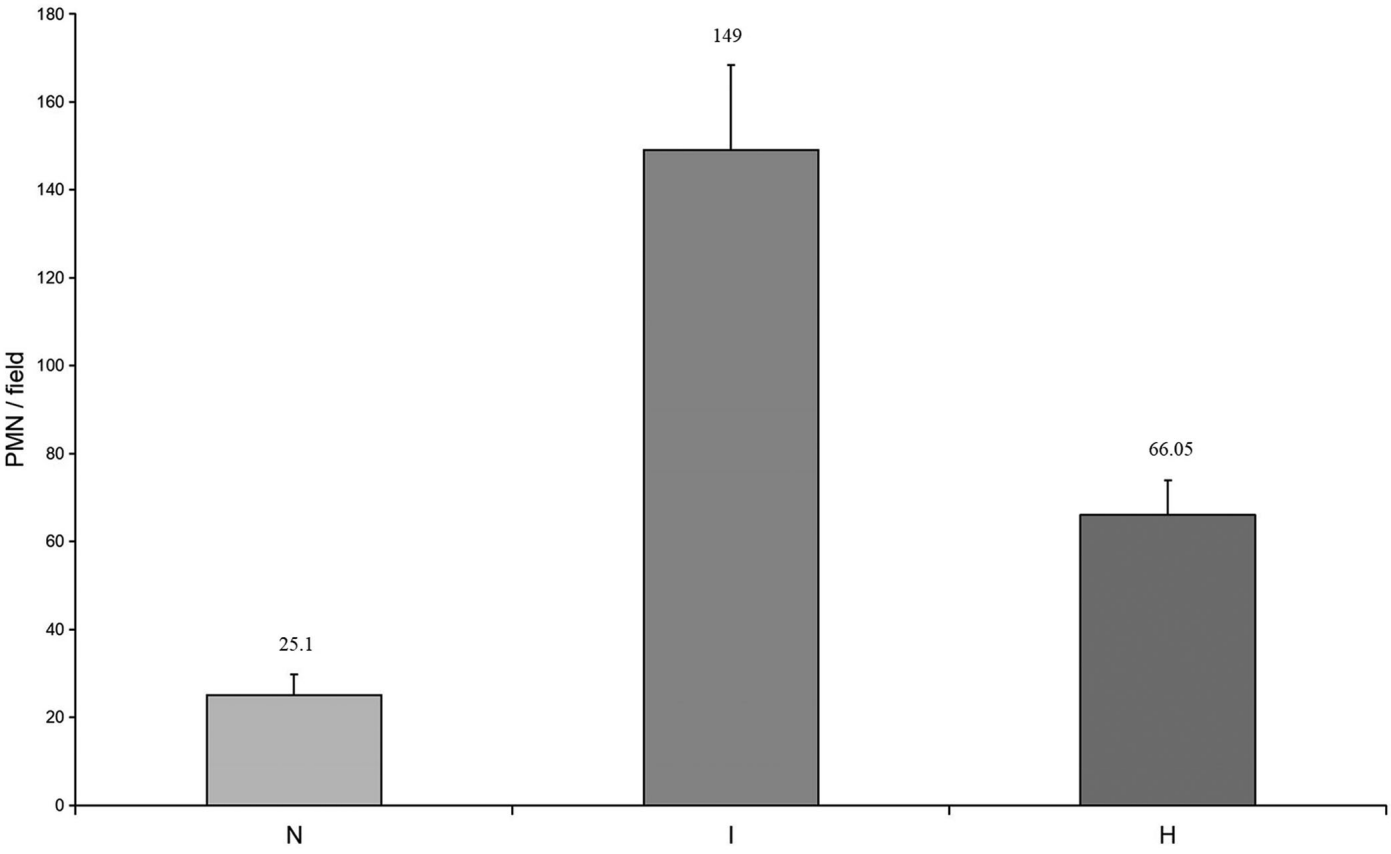
Number of neutrophils (PMNs) in the lung after IRI and CO_2_ inhalation. A rapid increase of neutrophils at 24 h in the I group is observed, and the increase versus that in the control group is significant. In the H group, the number decreases significantly compared with that in the I group. The number of cells is expressed per field of section. Data are expressed as the mean ± SD, bars represent SD, *p < .05, #*p* < .01, and six rats per group were examined.

The kinetics of neutrophil infiltration in the lung were evaluated by counting neutrophils in five fields in each rat. The number of neutrophils in the lung after IRI and CO2 inhalation were counted in the sections using light microscopy. Neutrophils were identified and located in the lung tissue; they adhered to the capillary vessels or were suspended in the pulmonary alveolus. The lungs of rats in the control group exhibited a few neutrophils, but the number sharply increased after transplantation, reaching almost 170 per field. This increase, compared with that of the control group (149 ± 19.32 vs. 25.1 ± 4.61; *p* < .01), was significant. After treatment with CO_2_ inhalation, the infiltration of neutrophils decreased dramatically, and infiltration was much lower than that in the IRI group (66.05 ± 7.8 vs. 149 ± 19.32; *p* < .05). However, this value was still higher than that of the control group (Figure 2).

### 3.4 Measurement of γδT cells

**Figure 3:**
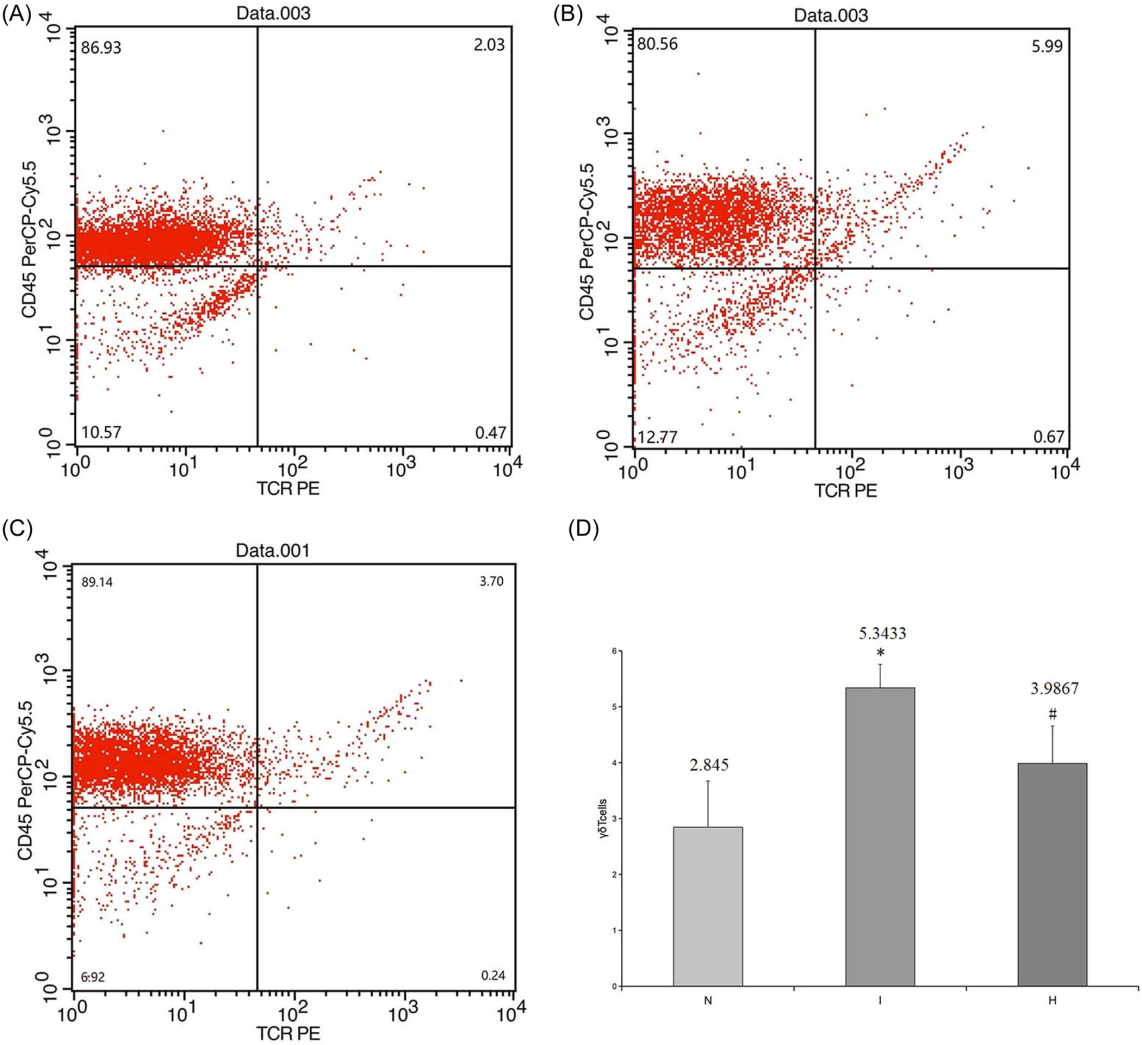
The number of γδT cells measured by flow cytometry. (A) Shows the representative graphs of γδT cell expression for each group; (B) the level of γδT cell expression significantly improves during IRI after 24 h compared with that of the control group; (C) treatment with CO_2_ can effectively decrease the expression of γδT cells. (D) Data are expressed as the mean ± SD, bars represent SD, *p < .05, #*p* < .01, and six rats per group were examined.

The expression of γδT cells was measured using flow cytometry. The level of γδ T cells significantly increased in group I compared to the control group (5.21 ± 0.51 vs. 2.85 ± 0.83; *p* < .01). Treatment with CO2 effectively decreased the expression of γδT cells, and this decrease was significant compared with that of the IRI group (3.99 ± 0.67 vs. 5.21 ± 0.51; *p* < .05), but the value was still higher than that in the control group (*p* > .05) (Figure 3).

### 3.5 Relationship between γδT cells and BALF IL‐17 or neutrophils

**Figure 4:**
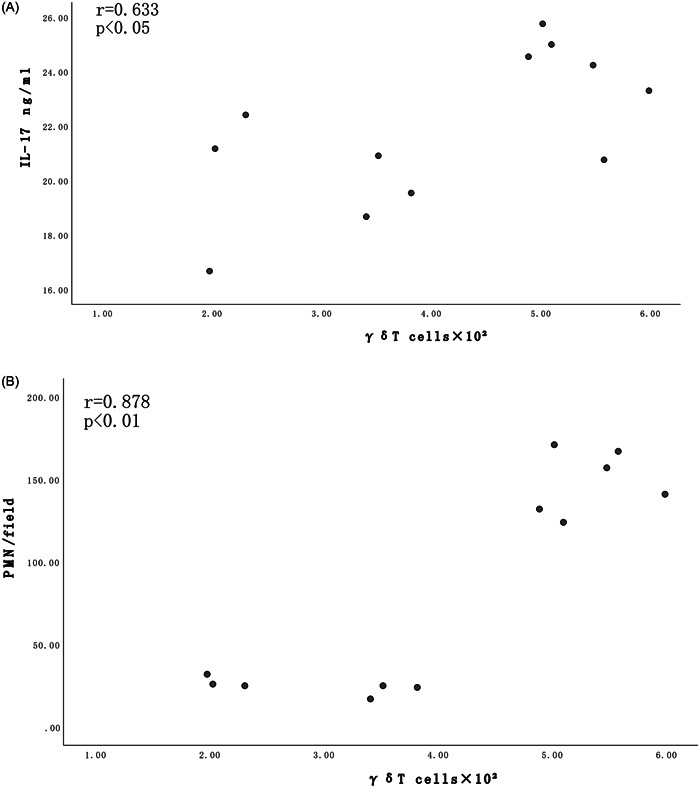
(A) Correlation between γδT cells and BALF IL‐17 levels; (B) correlation between γδT cells and/or neutrophils in the N and I groups. Increases in BALF IL‐17 and neutrophils are associated with increases in γδT cells.

As shown in Figure 4, a significant correlation was detected between γδT cells and BALF IL‐17 levels (*r* = .633, *p* < .05) or neutrophils (*r* = .878, *p* < .01) in the N group and I group. Increases in BALF IL‐17 and neutrophils were associated with increases in γδT cells (Figure 4).

### 3.6 Lung W/D weight ratio

The lung W/D weight ratio, used as an indicator of edema, was significantly increased in the IRI group versus the control group (6.45 ± 0.69 vs. 5.48 ± 0.15; *p* < .01). Importantly, the W/D weight ratio was significantly reduced in the H group compared with the control group (5.71 ± 0.15 vs. 6.45 ± 0.69; *p* < .05) (Table 1).

### 3.7 Histological examination

**Figure 5:**
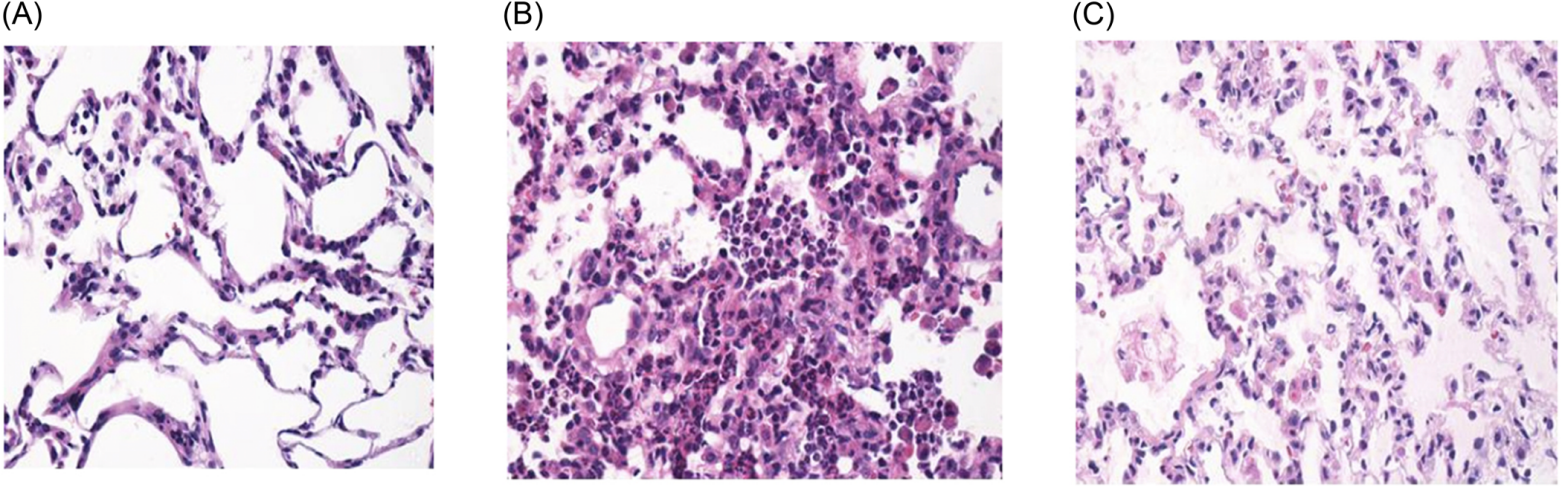
(A) In the control group, most of the alveolar walls are thin and complete, with only mild edema and occasional local neutrophil infiltration; (B) in the I group, more severe phenotypes are observed. Moderate to severe edema is observed in most of the alveolar septa and spaces, and more intensive neutrophil infiltration is observed; (C) in the H group, alveolar edema is alleviated, and the pulmonary characteristics are improved.

Grafts from the control group showed a relatively intact alveolar structure. Most of the alveolar walls were thin and complete, with only mild edema and occasional local neutrophil infiltration. Grafts from the IRI group showed more severe phenotypes than those from the control group. These grafts exhibited moderate to severe edema in most of the alveolar septa and spaces, much more intensive neutrophil infiltration, and alveolar hemorrhaging when compared to control grafts. Grafts from the H group showed that CO_2_ alleviated alveolar edema, ameliorated neutrophil infiltration, and effectively improved the pulmonary condition (Figure 5).

## 4 DISCUSSION

In the present study, we found that hypercapnia had a protective effect on lung transplantation since the expression of γδ T cells increased after IRI and decreased with hypercapnia treatment. Therapeutic hypercapnia downregulates γδ T cells, which can cause neutrophil infiltration and produce IL‐17, attenuating lung injury‐related IRI.

IRI is a critical medical condition that poses an important therapeutic challenge for physicians.^18^ The mechanisms of IRI are complex and multifactorial. The possible mechanisms suggested in previous studies include the generation of reactive oxygen and nitrogen species, the activation of leukocytes, and the dysfunction of the electron transport chain in mitochondria.^18^ IRI is unavoidable during lung transplantation and often leads to acute, sterile inflammation posttransplantation.^19^ Lung IRI‐induced inflammation can result in a rapid release of pro‐inflammatory mediators, such as tumor necrosis factor‐alpha, IL‐1β, and IL‐8, which contribute to pulmonary dysfunction.^19^, ^20^

IL‐17 has been known to be largely expressed by activated T cells and is involved in host innate immune responses. More importantly, numerous studies have provided evidence that IL‐17‐producing CD4+T cells play a critical role in organ rejection and IRI in the heart, brain, and liver.^8^, ^21^, ^22^ Data from the study conducted by Wang et al.^23^ demonstrated that IL‐17 was involved in the inflammatory response of IRI in acute kidney injury, and inhibition of IL‐17 might be a novel therapeutic strategy for the treatment of acute kidney injury. A finding of the study by Feng et al.^22^ was that inhibiting IL‐17 could alleviate cerebral IRI in aged mice. Wu et al.^24^ found that using triptolide to inhibit the production of IL‐17 could protect mice from IRI.

The above studies demonstrate the important role of IL‐17 in the development of IRI. But the mechanistic role of IL‐17 in IRI after lung transplantation is not well studied. In their study, Sharma et al.^6^ found that lung injury, inflammation, and neutrophil recruitment after IRI were highly dependent on the mechanism related to IL‐17. In this study, we measured IL‐17 expression in the early phase of lung IRI. As expected, IL‐17 in BALF and serum were elevated in the IRI group, which indicated that IL‐17 was involved in early IRI after lung transplantation.

γδ T cells are one of the major sources of IL‐17 in humans.^25^ It has been found that γδ T cells can produce IL‐17 in many different diseases or conditions.^25^ In this study, we detected a significant correlation between γδ T cells and BALF IL‐17 levels in lung IRI. Our results indicated that the secretion of IL‐17 was partially dependent on γδ T cells in lung IRI after lung transplantation, consistent with previous studies.^25^, ^26^ γδ T cells can combine features of the adaptive immune system, such as undergoing thymic selection and expression of a T cell receptor, initiating rapid immune responses.^27^

Several scholars have reported the potential role of γδ T cells in organ transplantation. In cerebral, myocardial, liver, and renal transplantation, γδ T cells have been shown to aggravate ischemic injury by generating chemotactic signals for peripheral myeloid cells such as neutrophils and monocytes.^28^, ^29^ In lung transplantation, Stankovic et al.^27^ found that γδ T cells were associated with cytomegalovirus replication. Consistent with the results of previous studies, we also found that γδ T cells were involved in lung IRI. The depletion of γδ T cells prolonged graft survival and reduced inflammatory cell infiltrates and mediator release. The function of γδ T cells on lung IRI could be related to their role in IL‐17 secretion.

Therapeutic hypercapnia is a ventilation strategy to allow for an unphysiological PaCO_2_ to permit lung‐protective ventilation with lower tidal volumes. It has been reported in a previous study that hypercapnia after lung IRI could inhibit T cells and decrease the level of pro‐inflammatory factors while increasing the level of anti‐inflammatory factors.^30^ Li et al.^31^ found that therapeutic hypercapnia could attenuate hepatic IRI through its effect on inflammation and apoptosis. Similarly, in Tan's study, hypercapnia was proven to regulate T lymphocytes and inhibit immune reactions, thus alleviating lung IRI.^14^ In the present study, we found that the levels of γδ T cells and IL‐17 were increased after IRI. However, therapeutic hypercapnia could effectively decrease the expression of γδ T cells and IL‐17. Furthermore, in our histologic examination, we found that CO_2_ treatment also alleviated alveolar edema, ameliorated neutrophil infiltration, and effectively improved pulmonary condition. Our results indicate that therapeutic hypercapnia could alleviate lung IRI after transplantation by inhibiting the expression of γδ T cells and IL‐17.

Our study has several limitations. First, our findings pertain to an animal model, which might not be completely transferable to a patient. However, the transfer of these models to human pathophysiology has been proven to be based on the same pathophysiologic pathways. Second, our data can be of value in explaining the injury of IL‐17‐producing γδ T cells and the meaningful advantages of therapeutic hypercapnia during lung transplantation. However, we did not detect the value of therapeutic hypercapnia in other interleukins. Finally, we did not investigate the direct relationship between γδ T cells and IL‐17 because of the conditional limitations of this study. We detected IL‐17 secretion in BALF and serum and made the correlation curve of γδ T cells and IL‐17. However, the existence of a clear direct relationship between γδT cells and IL‐17 can elucidate the function and effects of therapeutic hypercapnia described here, which needs to be probed further.

## 5 SIGNIFICANCE

Our results provide evidence of in vivo lung protection after lung transplantation. These data can be used to further enhance and guide the clinical acceptability of testing therapeutic hypercapnia as an option in critically ill patients if future mechanistic and translational studies confirm its benefits. Further work is required to untangle these conflicting phenotypes, discover activating ligands, and develop potential targets for therapeutic intervention.

## 6 CONCLUSION

We found that therapeutic hypercapnia provided defense against IRI after lung transplantation and reduced the expression of γδT cells, as well as decreased the inflammation response in lung tissue by regulating neutrophil activation and IL‐17 secretion, which play a vital role in the lung protection strategy.

## AUTHOR CONTRIBUTIONS

**Qing Xie**: Conceptualization; data curation; formal analysis; methodology; project administration; writing—review & editing. **Jia Lu**: Data curation; formal analysis; investigation; resources; supervision; validation; writing—original draft. **XiaoGuang Cui**: Conceptualization; methodology; resources; supervision; writing—review & editing.

## CONFLICT OF INTEREST STATEMENT

The authors declare no conflict of interest.

## ETHICS STATEMENT

All experiments were evaluated and approved by the ethics Committee of First Affiliated Hospital of Hainan Medical College and complied with the National Institutes of Health Guide for the Care and Use of Laboratory Animals.

## Data Availability

All data generated or analysed during this study are included in this article. Further enquiries can be directed to the corresponding author.
